# Biomolecules from Plant Residues

**DOI:** 10.3390/biom10111496

**Published:** 2020-10-30

**Authors:** Carmen Ancín-Azpilicueta, Irene Esparza, Nerea Jiménez-Moreno

**Affiliations:** Institute for Advanced Materials (INAMAT2), Department of Sciences, Universidad Pública de Navarra, 31006 Pamplona, Spain

The circular economy is a new model of production and consumption that involves reusing, renewing and recycling products to create added value. In this way, the life cycle of products is lengthened and waste is reduced to a minimum. As a consequence of this circular approach, the recovery of plant by-products has become increasingly important, providing multiple environmental, economic and social benefits, since their reuse generates new valuable products [1]. Moreover, this model reduces the negative consequences of resource scarcity and environmental degradation, unlike the linear economic model, based mainly on the "use and throw away" concept that requires large amounts of raw materials and energy [2].

Vegetable waste produced by the processing industry is a matter of great concern as it is generated in very large quantities [3]. Furthermore, current plant waste management strategies, in addition to being costly, have an adverse impact on the environment. These residues can cause high levels of environmental contamination, since they present a high biodegradability associated with high moisture content and microbial load. Anaerobic biological degradation of organic matter is the third anthropogenic source of atmospheric methane emissions, reaching ca. 32 million tons in 2010 worldwide, which represents the equivalent of 800 million tons of CO_2_ [4]. Therefore, there is a great need to develop strategies in order to reuse these plant by-products and turn them into useful products at the lowest possible cost [5]. The processing of fruits and vegetables, plants in general, produces several types of by-products such as solid residues of shells, peels, seeds, stems, pulp... that can constitute a good natural source of carbohydrates, proteins and bioactive molecules [6,7] for the food, cosmetic and pharmaceutical industries [8].

This Special Issue of *Biomolecules* is dedicated to current research on obtaining molecules with biological activity from plant residues and their subsequent use in the development of new products and/or applications. In this context, the waste valorization is the set of processes applied to the waste with the aim of exploiting the resources they contain, using them or transforming them to obtain energy (for example, liquid or gaseous fuels) or chemical products (both plat-form and more elaborated chemicals). The optimal valorization approach will depend on the nature and properties of the substances that are present or can be obtained from each type of residue. Currently, there are many studies on the composition of plant residues, which show that this type of waste constitutes a very important source of bioactive compounds (vitamins, minerals, antioxidants…). These biomolecules are essential and non-essential compounds found in nature and have been shown to have an effect on human health [9].

In this context, one of the first steps in the valorization of residues from vegetal origin is to obtain extracts rich in potential bioactive compounds. The present Special Issue collects different works that study the extraction method and the composition of extracts from different sources such as hazelnuts and walnuts shells, Goji (*Lycium barbarum* L.) leaves, Carménère pomace, tomato pomace and *Agave sisiana* agro-industrial residue. These are very different and varied plant origins, and all of them contained bioactive compounds with potential interest for different applications, which reveals the importance of the plant residues as relevant sources of biomolecules. 

For instance, Herrera et al. [10] analyzed qualitatively and quantitatively different extracts of hazelnut and walnut shells. These shells are a plentiful by-product of the edible nut processing industries and have traditionally been considered as a low-value waste. However, they are a source of valuable compounds with an interesting chemical profile for the chemical and pharmaceutical sectors. The experimental work included the isolation of crude lipophilic and hydrophilic extracts by means of an accelerated extraction process, different chromatographic analyses (Gas Chromatography–Flame Ionization Detection (GC–FID), GC Mass Spectroscopy (GC–MS), High-Pressure Size Exclusion Chromatography (HPSEC), Thin Layer Chromatography (TLC)) and the subsequent evaluation of the antioxidant capacity of these extracts. The lipophilic extracts were mainly composed of short chain fatty acids (stearic, palmitic and oleic acids), triglycerides and sterols. The more polar fractions presented a higher antioxidant capacity than the crude extracts.

Unlike nutshells, Goji berries (*Lycium barbarum* L.) have been widely described as a valuable source of bioactive compounds with great potential for the development of health-promoting formulations. The work of Conidi et al. [11] aimed to evaluate the potential of a combination of aqueous extraction and different purification operations based on different membranes for the recovery of phenolic compounds from Goji leaves, much less studied than its fruits. Among the analyzed membranes, a 1 kDa membrane exhibited the best result, eliminating part of the soluble solids and sugar compounds from polyphenols and improving the antioxidant activity of the aqueous extracts. 

Huamán-Castilla et al. [12] evaluated the improvement of polyphenols recovery from Carménère grape pomace by using glycerol as a co-solvent in aqueous extractions using a Hot Pressurized Liquid Extraction (HPLE) system. Grape pomace is very rich in different types of phenolic compounds, which are highly demanded for the production of functional ingredients for the food, nutraceutical, and cosmetic industries. These authors analyzed the influence on the extraction of low molecular weight polyphenols from different water–glycerol mixtures (15%, 32.5% and 50%) at 90, 120 and 150 °C. The optimal extraction conditions were 150 °C with 32.5% glycerol in the mixture for flavanols and with 50% glycerol for flavanols, stilbenes and phenolic acids. Another very interesting work related to the improvement of bioactive extraction processes is that of Nagarajan et al. [13]. These authors evaluated the potential of a recently developed water-glycerol induced complexation approach for the extraction of carotenoids from tomato pomace. Tomato pomace is a rich source of lycopene, β-carotene and pectin, which makes it an ideal by-product to optimize this type of extraction. The carotenoids and pectin recovered were structurally analyzed by High-Performance Liquid Chromatography (HPLC) and spectroscopy. The results obtained indicate that the carotenoid-pectin complex is a promising green approach to valorize agricultural by-products rich in carotenoids, valuable for their antioxidant properties.

Andrade et al. [14] demonstrated, both in vitro and in vivo, the potential of *Agave sisalana* agroindustrial residue as a safe and effective alternative for the prevention of the damage caused by oxidative stress and skin aging. This plant from Mexico, commonly known as sisal, has a great socioeconomic importance because it is the main source of hard fiber for the production of threads and ropes worldwide. More than 90% of the gross weight of *A. sisalana* leaves is discarded as agro-industrial residue with no commercial value, when it constitutes a promising source of biomolecules such as saponins and phenolic compounds. The antioxidant activity was evaluated in vitro (total antioxidant capacity, reducing power, DPPH radical scavenging, metal chelator (Fe^2+^ and Cu^2+^) and hydroxyl radical scavenging) and in vivo using the *Caenorhabditis elegans* organism model. The extract showed in vitro antioxidant activity in all cases, reduced the intracellular levels of reactive oxygen species (ROS) in *C. elegans* and increased the survival rate of these worms.

The contribution of Aliaño-Gonzalez et al. [15] to this Special Issue is an in-depth review of the composition and concentration of stilbenes in grapevine canes, which are the waste generated in the vineyard after the harvest. Different methods of stilbene extraction from grapevine canes and the extraction conditions are also compiled, highlighting the advantages and disadvantages of each technique. These stilbene-rich extracts have applications in several fields as preservatives, antifungals, insecticides or biostimulants. Likewise, Iriondo-DeHond et al. [16] present a review on the coffee processing by-products, analyzing their composition, safety and those food applications that have been proposed or commercialized to date. Dietary fiber is the main component of all coffee by-products and could easily be extracted for its use as a functional ingredient within the concept of a healthy diet. The shell and silver skin are also a potential source of micronutrients, vitamins and minerals, such as ascorbic acid and potassium. The main phenolic compounds found in these by-products are chlorogenic acids and anthocyanins with important antioxidant and antidiabetic properties, respectively.

This Special Issue also includes different works in which different recovery strategies and applications of extracts or products obtained from plant residues are proposed. Drozłowska et al. [17] showed the potential for the production of spray-dried functional powders with different emulsifying activity from flaxseed oil cake extract (residue from the production of cold-pressed flaxseed oil). Flaxseed oil cake is very rich in proteins and polysaccharides, so it has good emulsifying properties that could be used in the production of, for example, low-fat products. This work may open a promising pathway for the production of plant-based spray-dried powders for food applications as emulsion stabilizers. The work of Esparza et al. [18] studied the effect of the partial substitution of SO_2_ by a stem extract from Mazuelo grape and by a commercial extract of grapevine wood (Vinetan^®^) in Tempranillo wine. SO_2_ is a very important preservative for wine and other foods, but it has several drawbacks mainly associated with various allergic reactions. After 12 months of ageing in bottle, the wines obtained by adding extracts hardly showed any differences with respect to the control wines (with normal doses of SO_2_). Sensorial analysis revealed that the use of both extracts as partial substitutes for SO_2_ could lead to wines with good organoleptic properties, similar or even better than the control wines.

In the work of Lam et al. [19], the effects and potential of schisandrin B in combination with albendazole to treat *Angiostrongylus cantonensis*-induced meningoencephalitis was investigated. Currently, these infections are predominantly treated with albendazole, but the use of this anthelmintic can provoke certain neurological symptoms due to the immune response triggered by the dead worms. Therefore, treatment usually involves co-administration of corticosteroids to reduce the inflammatory reaction. Corticosteroids play a useful role in suppressing inflammation in the brain; however, long-term usage or high dosage may become problematic. Schisandrin B, an active component from *Schisandra chinensis*, has been shown to have anti-inflammatory effects on the brain. This study showed that albendazole-schysandrin B co-therapy could be used as a promising treatment for *Angiostrongylus*-induced meningoencephalitis.

Huge amounts of hemp core residues (43,000 tonnes) are collected annually in Europe with no significant application end. Such lignocellulosic wastes have potential as a filler or reinforcing material to replace synthetic fibers and wood fibers in polymeric composites. In the study by Vilaseca et al. [20], the hemp core biomass was treated with different concentrations of NaOH and then defibrated using a Sprout Waldron equipment to obtain individual fibers. The results showed that the flexural strength of the composite materials increased with the intensity of the NaOH treatment. The effect of NaOH was attributed to the removal of extracts and lignin in the cell wall of the fiber, which led to an improvement in the interfacial adhesion characteristics. Another work that shows the interest to take advantage of lignocellulosic materials is that of Cornejo et al [21], where these materials are proposed as promising alternatives to non-renewable fossil sources in order to produce aromatic compounds. These authors isolated and characterized *Populus salicaceae*, *Pinus radiata* and *Pinus pinaster* lignins from industrial waste and biorefinery effluents. Lignin was depolymerized using homogeneous (NaOH) and heterogeneous (Ni-, Cu- or Ni-Cu-hydrotalcites) base catalysis and catalytic hydrogenolysis using Ru/C. The poly-(hydroxy)-aromatic ether fraction was characterized by size exclusion chromatography (SEC), diffusion-ordered nuclear magnetic resonance spectroscopy (DOSY NMR), ^31^P NMR, and heteronuclear simple quantum coherence spectroscopy (HSQC). These analyses allowed us to establish the preeminent role of thermo-solvolysis processes in the depolymerization of lignin and showed that the synergistic effect of Ni and Cu provided monomers with oxidized alkyl side chains.

He et al. [22] obtained a ferulic acid dilactone from ferulic acid (FA) by means of a peroxidase-catalyzed radical coupling and made it react under different base/acid conditions. From the treatment with NaOH, new derivatives of this FA dimer such as 2-(4-hydroxy-3-methoxybenzylidene)-3-(hydroxyl-(4-hydroxy-3-methoxyphenyl)-methyl)-succinic acid and 2-(bis(4-hydroxy-3-methoxyphenyl)-methyl)-succinic acid were obtained. Additionally, a novel 8-8-coupled cyclic FA dimer (diethyl 6-hydroxy-1-(4-hydroxy-3-methoxyphenyl)-7-methoxy-1,2-dihydronaphthalene-2,3-dicarboxylate) was identified in products from FA dilactone treated by dry HCl in absolute ethanol. The synthesis of these diferulates is very important because they can serve as reference compounds to validate FA dimers isolated from plants for their potential as antioxidants and antimicrobials. Maznah et al. [23] carried out a field experiment to investigate the degradation of metsulfuron-methyl applied to an oil palm plantation in two different doses. Soil samples were collected at different depths (0–10, 10–20, 20–30, 30–40 and 40–50 cm), and different days after the treatment. The results showed a rapid degradation of metsulfuron-methyl in the soil, with calculated values of half-life (t½) ranging between 6.3 and 7.9 days

Altogether, the works included in this Special Issue illustrate examples of the recent progress on the technological advances and applications of different approaches to obtain bioactive substances or high value-added products from plant residues. The general objective was to compile research and review articles that cover the latest trends in the area and thus benefit researchers and readers in advancing their knowledge on the subject.

## References

[1] Coderoni S., Perito M.A. (2020). Sustainable consumption in the circular economy. An analysis of consumers’purchase intentions for waste-to-value food. J. Clean. Prod..

[2] Morseletto P. (2020). Targets for a circular economy. Resour. Conserv. Recycl..

[3] Banerjee J., Singh R., Vijayaraghavan R., MacFarlane D., Patti A.F., Arora A. (2017). Bioactives from fruit processing wastes: Green approaches to valuable chemicals. Food Chem..

[4] Breeze P., Breeze P. (2018). Landfill waste disposal, anaerobic digestion, and energy production. Energy From Waste.

[5] Esparza I., Jiménez-Moreno N., Bimbela F., Ancín-Azpilicueta C., Gandía L.M. (2020). Fruit and vegetable waste management: Conventional and emerging approaches. J. Environ. Manag..

[6] Grigoras C.G., Destandau E., Lazãr G., Elfakir C. (2012). Bioactive compounds extraction from pomace of four apple varieties. J. Eng. Stud. Res..

[7] Kowalska H., Czajkowska K., Cichowska J., Lenart A. (2017). What’s new in biopotential of fruit and vegetable by-products applied in the food processing industry. Trends Food Sci. Technol..

[8] Jiménez-Moreno N., Esparza I., Bimbela F., Gandía L.M., Ancín-Azpilicueta C. (2020). Valorization of selected fruit and vegetable wastes as bioactive compounds: Opportunities and challenges. Crit. Rev. Environ. Sci. Tec..

[9] Biesalski H.K., Dragsted L.O., Elmadfa I., Grossklaus R., Műller M., Schrenk D., Weber P. (2009). Bioactive compounds: Definition and assessment of activity. Nutrition.

[10] Herrera R., Hemming J., Smeds A., Gordobil O., Stefan Willför S., Labidi J. (2020). Recovery of bioactive compounds from hazelnuts and walnuts shells: Quantitative–qualitative analysis and chromatographic purification. Biomolecules.

[11] Conidi C., Drioli E., Cassano A. (2020). Biologically active compounds from Goji (*Lyciumbarbarum* L.) leaves aqueous extracts: Purification and concentration by membrane processes. Biomolecules.

[12] Huamán-Castilla N.L., Mariotti-Celis M.S., Martínez-Cifuentes M., Pérez-Correa J.R. (2020). Glycerol as alternative co-solvent for water extraction of polyphenols from Carménère pomace: Hot pressurized liquid extraction and computational chemistry calculations. Biomolecules.

[13] Nagarajan J., Kay H.P., Krishnamurthy N.P., Ramakrishnan N.R., Aldawoud T.M.S., Galanakis C.M., Wei O.C. (2020). Extraction of carotenoids from tomato pomace via water-induced hydrocolloidal complexation. Biomolecules.

[14] Andrade Gomes Barreto S.M., Muñoz Cadavid C.O., De Oliveira Moura R.A., Martins Silva G.M., Ferreira de Araújo S.V., Aderaldo da Silva Filho J.A., Oliveira Rocha H.A., De Paula Oliveira R., Brandt Giordani R., Ferrari M. (2020). In vitro and in vivo antioxidant activity of Agave sisalana agro-industrial residue. Biomolecules.

[15] Aliaño-González M.J., Richard T., Cantos-Villar E. (2020). Grapevine cane extracts: Raw plant material, extraction methods, quantification, and applications. Biomolecules.

[16] Iriondo-DeHond A., Iriondo-DeHond M., Del Castillo M.D. (2020). Applications of compounds from coffee processing by-products. Biomolecules.

[17] Drozłowska E., Łopusiewicz Ł., Mȩżyńska M., Bartkowiak A. (2020). Valorization of flaxseed oil cake residual from cold-press oil production as a material for preparation of spray-dried functional powders for food applications as emulsion stabilizers. Biomolecules.

[18] Esparza I., Martínez-Inda B., Cimminelli M.J., Jimeno-Mendoza M.C., Moler J.A., Jiménez-Moreno N., Ancín-Azpilicueta C. (2020). Reducing SO2 doses in red wines by using grape stem extracts as antioxidants. Biomolecules.

[19] Lam H.Y.P., Liang T.R., Jiang S.J., Peng S.Y. (2020). Albendazole-schisandrin B co-therapy on Angiostrongylus cantonensis-induced meningoencephalitis in mice. Biomolecules.

[20] Vilaseca F., Serra-Parareda F., Espinosa E., Rodríguez A., Mutjé P., Delgado-Aguilar M. (2020). Valorization of hemp core residues: Impact of NaOH treatment on the flexural strength of PP composites and intrinsic flexural strength of hemp core fibers. Biomolecules.

[21] Cornejo A., Bimbela F., Moreira R., Hablich K., García-Yoldi I., Maisterra M., Portugal A., Gandía L.M., Martínez-Merino V. (2020). Production of aromatic compounds by catalytic depolymerization of technical and downstream biorefinery lignins. Biomolecules.

[22] He Y., Jia Y., Lu F. (2020). New products generated from the transformations of ferulic acid dilactone. Biomolecules.

[23] Maznah Z., Ismail B.S., Eng O.K. (2020). Residue and dissipation kinetics of metsulfuron-methyl herbicide in soil: A field assessment at an oil palm plantation. Biomolecules.

